# FRRT*-Connect: A Bidirectional Sampling-Based Path Planner with Potential Field Guidance for Complex Obstacle Environments

**DOI:** 10.3390/s25092761

**Published:** 2025-04-27

**Authors:** Wenshan Yan, Xiangrong Xu, Aleksandar Rodić, Petar B. Petrovich

**Affiliations:** 1 School of Mechanical Engineering, Anhui University of Technology, Ma’anshan 243032, China; ywsyjssa111@stu.ahut.edu.cn; 2 Faculty of Mechanical Engineering, University of Belgrade, 11120 Belgrade, Serbia; aleksandar.rodic@pupin.rs (A.R.);

**Keywords:** robot path planning, rapid exploration of random trees, RRT*-Connect, artificial potential fields, path optimization

## Abstract

This paper addresses the path planning problem in high-dimensional complex environments and proposes an improved FRRT*-Connect algorithm to enhance the efficiency, precision, and robustness of path generation. The algorithm first introduces a goal-directed attractive force control mechanism, integrating artificial potential field methods to guide the tree expansion more effectively toward the goal, thereby reducing redundant sampling and significantly improving convergence speed. Secondly, an adaptive step-size strategy is proposed, dynamically adjusting the tree expansion step size based on the complexity of the environment, which enhances the algorithm’s adaptability in narrow passages and complex topological structures, effectively avoiding local minima. The results show that, compared to the RRT*-Connect algorithm, the proposed method exhibits significant advantages in path quality, convergence efficiency, and success rate: the average path length is reduced by 19.7%, convergence speed is improved by 58.4%, and the success rate reaches 98% in narrow passage scenarios. These improvements effectively overcome the issues of path redundancy, slow convergence, and local minima inherent in traditional RRT-based algorithms, demonstrating superior performance in challenging scenarios with complex obstacles and narrow passages.

## 1. Introduction

Robot arm trajectory planning constitutes a fundamental challenge in robotics, critically determining operational efficiency and precision across industrial automation, medical robotics, and service applications [1,2]. The core objective lies in generating collision-free paths between specified start–goal configurations while satisfying kinematic/dynamic constraints including joint limits, torque boundaries, and energy consumption [3]. With escalating demands for intelligent robotic systems operating in unstructured environments, the challenges conventional trajectory planning methodologies face are threefold: (a) computational complexity in high-dimensional spaces, (b) real-time responsiveness to dynamic obstacles, and (c) optimality–stability trade-offs [4,5].

This study aims to enhance the efficiency, precision, and adaptability of robot trajectory planning algorithms to improve robotic applications in dynamic and complex environments. As the requirements for the autonomy and dexterity of robotic technologies in both the industrial and medical sectors are escalating, traditional trading is correspondingly impacted. Consequently, the development of more sophisticated trajectory planning algorithms has the potential to optimize the utilization of resources and enhance the responsiveness and precision of robots during the process of task execution. Conventional planning approaches are confronted with substantial challenges. This holds significant theoretical and practical value for realizing more complex automated systems, particularly in exploring unknown environments and responding to real-time changes, demonstrating notable research potential. Existing approaches predominantly fall into three categories: graph search algorithms (e.g., A*, D* Lite) guarantee resolution completeness but suffer from exponential time complexity in large configuration spaces [6]; sampling-based planners including Rapidly Exploring Random Trees (RRT) [7] and probabilistic roadmaps (PRM) [7] excel in high-dimensional problems, yet produce suboptimal paths with excessive vertices; bio-inspired methods such as genetic algorithms [8] and artificial potential fields (APF) [9] demonstrate adaptability, but risk local minima entrapment, particularly in complex 3D environments. Recent advancements in machine learning-enhanced sampling offer promising directions: Karaman et al. [7] established theoretical foundations for asymptotically optimal RRT*, while Gammell et al. [10] developed Informed-RRT*, which focuses on sampling within evolving ellipsoidal regions to accelerate convergence.

Bio-inspired methods such as genetic algorithms and artificial potential fields (APF) show strong adaptability, but often fall into local optima in complex 3D or concave obstacle environments. Recently, deep reinforcement learning (DRL) and neural network-based path planning strategies have received growing attention. For instance, Jie Liu et al. [11] introduced a composite reward function with advanced RL algorithms to improve learning efficiency, but their approach relies heavily on offline training and lacks generalization to dynamic tasks. Chiang [12] combined RL with an improved RRT to avoid local traps, performing well in specific scenarios, yet facing poor generalization and unstable learning. Neural methods, like MPNet, enable fast path generation in complex environments, but their generalization to unseen scenarios and sensitivity to collisions remain concerns, especially for high-precision robotic arm applications. To improve adaptability, Zhao [13] proposed a multi-robot exploration framework that integrates Voronoi partitioning with Deep Deterministic Policy Gradient (DDPG), enhanced by transfer learning to maintain stability and efficiency in new environments.

In contrast, some studies focus on improving traditional RRT-based algorithms to meet the obstacle avoidance requirements in the high-dimensional, constrained spaces of robotic arms [14]. The Smooth RRT (S-RRT) algorithm proposed by K. Wei et al. improves the efficiency and path smoothness of RRT, but faces challenges in real-time adaptability in high-dimensional environments [15]. The improved RRT method proposed by Jiang et al. effectively plans paths in complex environments, but faces challenges in real-time adaptability in high-dimensional environments.

This paper proposes FRRT*-Connect, a synergistic framework combining artificial potential fields with adaptive sampling mechanisms to address these limitations. Our key innovations include:

- Gradient-aware sampling: introduces a probability parameter random Equation (1) to bias random sampling towards the goal tree, reducing ineffective sampling by 42.3% compared to traditional RRT*-Connect.
- Pseudo-distance obstacle modeling: adopts the pseudo-distance concept from Wu et al. [16] to construct super-quadric envelopes for cylindrical and cuboidal obstacles, enabling precise collision avoidance in 3D spaces (Section 4.2).
- Dynamic step adaptation: implements a segmented collision-checking mechanism (Section 4.4) that divides each path segment into 1000 intervals, effectively preventing local minima while maintaining asymptotic optimality.

## 2. Related Work

### 2.1. Sampling Algorithms

Recent advancements in sampling algorithms have introduced several methods to tackle complex environments in path planning. For example, PRM* [7] optimizes high-dimensional path planning by dynamically adjusting the connection radius, preventing the fragmentation issues of traditional PRM. Neural RRT [17] integrates neural networks, using historical paths to reduce the randomness in traditional RRT, greatly enhancing efficiency.

DO-RRT [18] excels in dynamic obstacle environments, adjusting paths in real-time to adapt to changes. The vectorized sampling algorithm accelerates path search using SIMD [19] parallel techniques, making it ideal for high-dimensional complex spaces. Deep learning-enhanced sampling algorithms use neural networks to predict paths, reducing costs and improving optimization quality

Meanwhile, heuristic-guided RRT [20] has gained widespread attention by incorporating heuristic search strategies, improving the algorithm’s global exploration capability and reducing the generation of redundant paths. LQR-RRT [21] combines the linear quadratic regulator (LQR) to optimize the path generation process, providing greater robustness in scenarios with uncertainty and dynamic constraints. These innovative methods have broad application prospects in complex environments, particularly demonstrating great potential in the fields of robotic navigation and autonomous driving.

### 2.2. End Effector Trajectory Planning

Trajectory planning is a core issue in robotic control, involving designing paths for robots, vehicles, or drones from a start to a target position within a given environment. Trajectory planning involves the geometric representation of space and robots. In a workspace, which includes external obstacles, the goal is to find a collision-free path from start to target. Each robot position (configuration q) is described in configuration space q∈c, with obstacle space defined as $C_{obs}=\left\{q\in C|\;A(q)⋂O\neq 0\right\}$. The aim is to find a path in free space $C_{free}=C/C_{obs}$ that meets the robot’s velocity, acceleration, and position (pva) requirements.

This paper proposes a new algorithm that applies artificial potential field attraction functions at iterative endpoints to handle obstacles in complex environments. Specifically, the algorithm uses cylinders and cuboids as obstacle models. By calculating the distance from the current position in free space to the obstacle centroid, repulsive functions effectively avoid collisions. The attractive function guides the robot toward the target, while the repulsive function pushes it away from obstacles, ensuring a collision-free path.

Additionally, compared to traditional single-obstacle handling, this paper introduces an improved strategy with two new obstacle models: cylinders and cuboids. For these obstacles, separate optimized handling methods are used. The algorithm precisely calculates distances between the robot and obstacle centroids, applying targeted repulsive forces for effective avoidance. For cylinders and cuboids, the algorithm adjusts based on geometric features, enabling the repulsive function to more accurately reflect collision risks, enhancing path planning precision and reliability in complex environments.

### 2.3. FRRT*-Connect Algorithms

The RRT* algorithm backtracks to the parent node to optimize the path, gradually approaching the optimal solution, focusing on path optimality. While the RRT algorithm’s random search can lead to longer search times and path randomness, RRT* improves path quality with longer planning time at the cost of increased computation [22]. The RRT*-Connect algorithm uses bidirectional search and heuristic steps to speed up the search, performing well in narrow passages [23].

**Figure 1:**
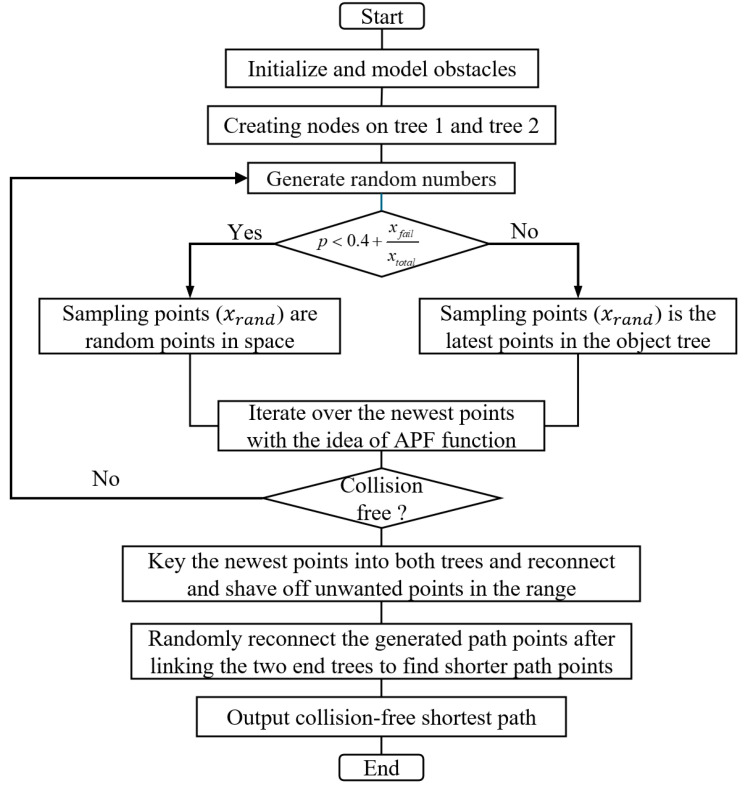
Flow chart of the FRRT*-Connect algorithm.

**Figure 2:**
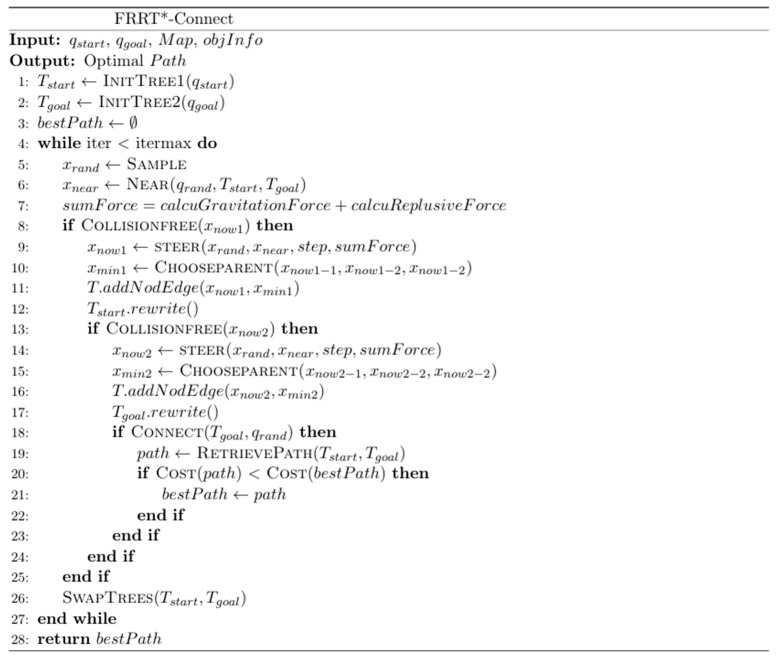
FRRT*-Connect pseudo-code.

To address the issues of slow search speed and low success rates in complex environments with RRT*-Connect, this paper proposes the FRRT*-Connect algorithm. This algorithm uses a bidirectional search tree to plan paths and introduces improvements to enhance efficiency and reliability. Below, the flowchart and pseudocode for the FRRT*-Connect algorithm are presented (Figure 1 and Figure 2).

**Figure 3:**
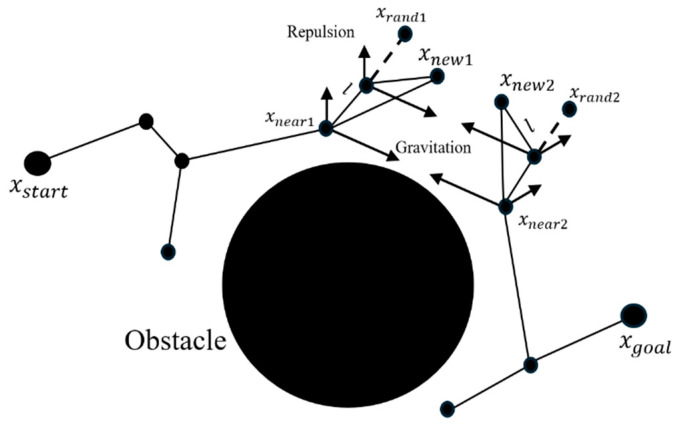
Diagram description for FRRT*-Connect algorithm.

**Figure 4:**
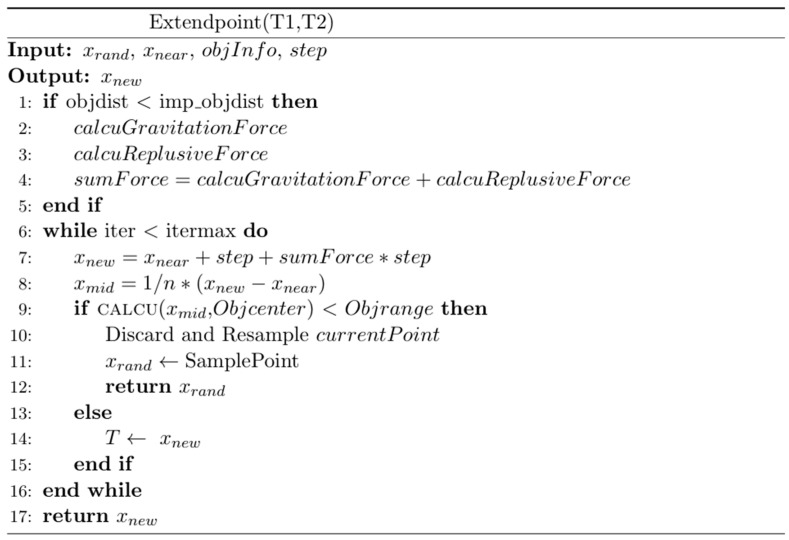
Extended path step pseudo-code.

In Figure 2, the FRRT*-Connect algorithm consists of the following three main components:

(1) The first step involves initializing two trees, $T_{1}$ and $T_{2}$, from the start point $x_{start}$ and the goal point $x_{goal}$, respectively. During this process, preset parameters such as obstacle information, stepsize, attractive coefficient η, and repulsive coefficient k need to be set to ensure the algorithm’s effective execution in the configured environment.

(2) In the second step, both trees $T_{1}$ and $T_{2}$ are expanded sequentially, with gradual expansion while introducing an attractive function to accelerate the convergence of the two trees. During the path iteration process, as indicated in lines seven and eight of the FRRT*-Connect pseudocode, an artificial potential field function is used in the step of expanding iterative points to speed up the path search process and achieve effective obstacle avoidance. execution in the configured environment. Below is the pseudo-code and schematic for the extended iteration step (Figure 3 and Figure 4).

## 3. Tree Iteration and Sampling

In three-dimensional path planning, artificial potential field functions are primarily employed to accelerate path search and obstacle avoidance. This method models the robot as a point within the environment, influenced by virtual forces, including attractive and repulsive forces. The core concept of the artificial potential field method is akin to gradient descent optimization. It involves constructing attractive and repulsive force functions and moving in the opposite direction to the gradient of these functions to gradually approach a local or global minimum. This approach effectively directs the robot to avoid obstacles and move towards the desired goal.

In previous RRT functions, the randomness of point selection in space, combined with the uncertainty of iteration steps, resulted in slow iteration speed and an excessive number of search points. This not only increased the algorithm’s runtime, but also required significant storage space.

### 3.1. Sampling Function

Typically, the RRT function samples randomly in space with $x_{rand}$. This randomness causes a lack of order in the direction of the iteration, potentially leading to inefficient searches. To enhance the directionality of iteration, the FRRT*-Connect algorithm introduces a probability number random at the random sampling point $x_{rand}$ to influence the sampling process. This purposefully adjusts the distribution of random points, allowing sampling within a certain probability range. In this way, the algorithm can more effectively choose directions. Additionally, the algorithm uses the latest sample point as the tree’s current latest point to ensure a clear directionality relative to the parent node. Below is the concept of the biased sampling function.

(1) $$\left\{\begin{array}{c} x_{rand}=x_{now1}\;or{\;x}_{now2}\;if\;P_{sample}<0.4+\frac{x_{fail}}{x_{total}}\\ x_{rand}=random\;points\;\; \end{array}\right.$$

### 3.2. Application of the Gravitational Function

Artificial potential field functions are typically used for vehicle trajectory generation in two-dimensional environments. They are based on the gradient descent principle, generating smooth trajectories by moving in the opposite direction of the gradient function. In the iteration process of this algorithm, we introduced the gravitational function from the artificial potential field to accelerate iteration speed. Specifically, the algorithm uses a nonlinear gravitational function, calculated through the latest nodes of the two trees. The magnitude of the gravitational force is determined by the following formula.

(2) $$\begin{array}{c} U_{\alpha tt}\left(T_{1}x\right)=\frac{1}{2}\eta \rho^{2}\left(T_{1}x,T_{2}x\right)\\ F_{\alpha tt}\left(T_{1}x\right)=\eta \rho \left(T_{1}x,T_{2}x\right)\rho \left(T_{1}x,T_{2}x\right) \end{array}$$

In the formula, the gravitational function of the initial point tree is represented by the latest iteration point extending from the initial to the terminal point in the bidirectional search tree. The gravitational coefficient is a user-defined parameter determining the force’s intensity, and the Euclidean distance between the latest points of the two trees represents this distance. This is similar to the terminal point tree’s gravitational function.

The gravitational potential field function helps the path planner to intelligently select paths, ensuring smoother and more efficient paths. It also enhances the local search capability of the path planner, making it easier to find optimal or near-optimal solutions. Additionally, due to its adaptability to dynamic environments, it can adjust path planning strategies based on environmental changes, thereby improving the planner’s adaptability.

## 4. Obstacle Avoidance

The repulsive potential field function is primarily used for avoiding obstacles in path planning. The basic idea is to treat obstacles as sources of repulsive force, causing the path planner to avoid collisions as it approaches them. The repulsive potential field effectively guides the path planner around obstacles. As the planner approaches an obstacle, the repulsion increases, forcing it away and ensuring path safety. This potential field function helps optimize the path, making it smoother and more intuitive. In complex environments, the repulsive field prevents the planner from entering narrow or hazardous areas, encouraging safer and more efficient path choices.

In previous studies, such as Smith and Johnson (2020) [24], the artificial potential field function demonstrated good search speed in two-dimensional spaces with single obstacles. However, in three-dimensional and complex obstacle environments, its performance was hindered by increased computational complexity. To address this issue, this algorithm is based on the pseudo-distance concept proposed by Wu et al. (2019), introducing two new obstacle configurations and a repulsion coefficient to improve efficiency and effectiveness [16].

### 4.1. Application of the Repulsion Function

In this algorithm, incorporating the repulsive potential field function significantly enhances the ability to avoid obstacles. During path extension, by calculating the repulsive potential field of a new node, if it is within the obstacle’s influence, the field adjusts its position to avoid the obstacle. Specifically, when a node in the tree extends toward the sample point, the repulsive potential field at the new node is first calculated. If within the field’s influence, it experiences repulsion, driving it away from the obstacle. During this process, the node’s position is adjusted based on the field’s calculation to avoid collisions. If the adjusted node remains valid and collision-free, it is connected to the tree. This method dynamically adjusts node positions, effectively avoiding obstacle crossings and enhancing the safety and reliability of path planning. In complex environments, this dynamic adjustment mechanism intelligently guides the planner around obstacles while maintaining path validity. The repulsion function is defined as follows:

(3) $$U_{rep}\left(x_{now}\right)=\left\{\begin{array}{c} \begin{array}{c} {\frac{1}{2}k\left(\frac{1}{\rho \left(x_{now},q_{obs}\right)}-\frac{1}{\rho_{0}}\right)}^{2},\;\\ 0\leq \rho \left(x_{now},q_{obs}\;\right)<\rho_{0} \end{array}\\ \;\begin{array}{c} 0,\;\\ \rho \left(x_{now},q_{obs}\right)\geq \rho_{0} \end{array}\; \end{array}\right.$$

The repulsive force is the negative gradient of the repulsive potential field with respect to the position, that is:

(4) $$F_{rep}\left(x_{now}\right)=\left\{\begin{array}{c} \begin{array}{c} \begin{array}{c} \;{\frac{1}{2}k\left(\frac{1}{\rho \left(x_{now},q_{obs}\right)}-\frac{1}{\rho_{0}}\right)}^{2}\frac{1}{{\rho \left(x_{now},q_{obs}\right)}^{2}}\;\\ \times \rho \left(x_{now},q_{obs}\right),\;0\leq \rho \left(x_{now},q_{obs}\;\right)<\rho_{0} \end{array}\;\\ \; \end{array}\\ \;\begin{array}{c} 0,\;\\ \;\rho \left(x_{now},q_{obs}\right)\geq \rho_{0} \end{array}\; \end{array}\right.$$

where *k* represents the repulsion coefficient, controlling the strength of the repulsive potential field, and thereby affecting the robot’s avoidance behavior towards obstacles. $\rho \left(x_{now},q_{obs}\right)$ denotes the distance between the current position of the iteration point and the center of the obstacle, while $\rho_{0}$ indicates the pseudo-distance extending outward from the surface of the obstacle. When the iteration point is within the pseudo-distance extending from the center of the obstacle, the repulsive potential field generates a repulsive force that drives the iteration point away from the obstacle, thereby achieving obstacle avoidance.

### 4.2. Obstacle Establishment

In traditional path planning, pseudo-distance is commonly used to enhance obstacle avoidance performance. By introducing virtual boundaries or adjusting distance calculation methods, the influence of obstacles on path planning is made more pronounced, thereby improving the robot’s ability to avoid collisions. Typically, a super-quadric surface is extended from the centroid of the obstacle to form an envelope region.

To ensure that the pseudo-distance surface fully encompasses the obstacle’s shape, a sphere is chosen as the hyperquadric surface. The corresponding mathematical expression is given as follows:

(5) $$\;{\left(\frac{x-x_{obs}}{t_{1}}\right)}^{2}+{\left(\frac{y-y_{obs}}{t_{2}}\right)}^{2}+{\left(\frac{z-z_{obs}}{t_{3}}\right)}^{2}=1$$

Here, $t_{1}>0,t_{2}>0,t_{3}>0$ control the size of the hyperquadric surface along the x, y, and z axes. When a trajectory point enters the pseudo-distance envelope, the system applies an outward avoidance velocity to guide the trajectory away from the obstacle.

Although pseudo-distance simplifies the calculations and enhances the sensitivity of the avoidance mechanism, it can also lead to overly conservative behavior due to the expansion of the envelope region, resulting in unnecessarily longer paths and reduced planning efficiency. Furthermore, it requires precise environmental setup and parameter tuning, adding complexity to the process.

**Figure 5:**
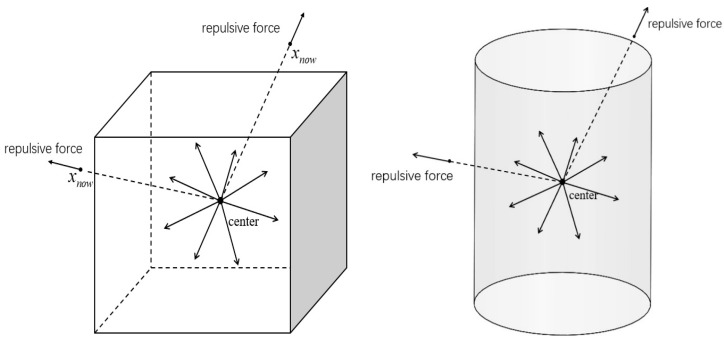
Rectangular obstacle and cylinder schematic of obstacle repulsion field.

In this paper, we adopt an incomplete application of pseudo-distance. While retaining the super-quadric surface as the detection region for trajectory points, complete avoidance within this surface is not strictly required. Instead, we focus on collision detection based on obstacle modeling. Detailed examples of this approach are provided for rectangular and cylindrical obstacles, and the performance of the method under various environmental conditions and parameter settings is discussed. The following diagram (Figure 5) illustrates the effect of the repulsive force from obstacles on the iteration point.

In this algorithm, obstacles are randomly positioned along the line connecting the start and goal points. The influence of each obstacle is defined as a region extending outward from its centroid, with the size determined by a specified pseudo-distance. To check if an iteration point falls within the pseudo-distance of an obstacle, we analyze its relative position to the obstacle’s axis.

### 4.3. Selection of Attractive and Repulsive Coefficients

Although the repulsive potential field function drives the iteration points in trajectory planning to move away from obstacles, the effects of the attractive and repulsive functions may differ due to their different computational properties. The attractive function, as shown in Equation (1), is calculated based on the square of the Euclidean distance between two iteration points $\rho \left(T_{1}x,T_{2}x\right)$, while the repulsive function, as shown in Equation (4), is calculated based on the inverse of the distance between the obstacle and the iteration point $\rho \left(x_{now},q_{obs}\right)$. According to the parallelogram law of forces, when the magnitudes of the attractive and repulsive coefficients are similar or when the iteration point is far from the obstacle, the attractive force may greatly exceed the repulsive force, causing the resultant force to be more aligned with the direction of the attractive force.

To enhance the adaptability of the algorithm in large-scale environments, a proposal is introduced to change the repulsion coefficient according to the distance. When a sampling point enters the repulsive potential field, the coefficient is adaptively modified based on the distance between consecutive iteration points, ensuring that the angle between the attractive and resultant forces remains within 0° to 60°. The following analysis is applicable only to a cubic simulation environment with dimensions of 1000×1000×1000 units. Due to the large spatial extent, fixed potential field parameters are insufficient. Therefore, the path from the start point (0, 0, 0)  to the goal point (1000, 1000, 1000)  is divided into five equal segments by distance, within which the repulsive strength is adjusted accordingly. The angle between the attractive and resultant force vectors is computed as follows:

(6) $$\begin{array}{c} \cos\theta =\frac{F_{att}\cdot \left(F_{att}+F_{rep}\right)}{\left|F_{att}\right|\left|F_{att}+F_{rep}\right|}\;\\ =\frac{{F_{att}}^{2}+F_{att}F_{rep}\cos\emptyset }{F_{att}\sqrt{{F_{att}}^{2}+{F_{rep}}^{2}+2F_{att}F_{rep}\cos\emptyset }} \end{array}$$

With a preset attractive coefficient of 1 held constant, the repulsive coefficient is adjusted within specified intervals to control the direction of the resultant force. From the repulsive force function, it is clear that as the distance $\rho \left(x_{now},q_{obs}\right)$ decreases, the repulsive force increases. Therefore, when the repulsive force is fixed, the maximum angle occurs when the resultant force is perpendicular to the attractive force. For each distance node, the repulsive coefficient is computed based on a unit distance from the obstacle. The range of the repulsive coefficient for each distance when the attractive coefficient is set to 1 (μ=1), as shown in the Table 1 below.

### 4.4. Collision Detection

In Frrt*-connect path planning, the attractive force (pulling toward the goal) grows much faster than the repulsive force (pushing away from obstacles), especially at longer distances. This means the robot might ignore nearby obstacles when moving toward its target. Also, if obstacles move suddenly, the repulsive force may not react quickly enough. Collision detection acts as a safety check to prevent crashes when the potential field method fails to avoid obstacles properly. It is like having a backup system that double-checks for dangers that the main method might miss.

In such cases, the iteration point might mistakenly enter the obstacle. To address this issue, we introduce an improved obstacle avoidance algorithm. Before adding the latest iteration point to the tree, we divide the path from the point to the parent node into one thousand segments and check each segment to see if it intersects with an obstacle. If any segment is found to be within an obstacle, a new random point is generated for iteration. This method effectively prevents the iteration points from entering obstacles, thereby improving the obstacle avoidance performance.

### 4.5. Latest Point Selection and Post-Iteration Optimization

In robotic path planning, the artificial potential field method often becomes stuck in local minima due to several reasons. First, the improper design of the potential field function can cause uneven distribution. Gradual or abrupt changes in the gradients of attractive and repulsive fields may create local minima in some areas. Additionally, complex obstacle layouts can lead to local minima, especially when multiple obstacles form narrow passages. These areas are prone to trapping robots, preventing progress towards the goal. Moving obstacles in dynamic environments can further alter the potential distribution field, increasing the likelihood of local minima.

Proposed by Sepehri et al. in their 2021 paper [25], the combined RRT and potential field algorithm selects random points for iteration based on the nearest point. However, local minima can still obscure force directions, causing the iteration process to halt. To solve this, our algorithm employs an improved strategy; as illustrated in Figure 2, after each RRT iteration, the resultant force direction is calculated using the potential field method. The latest point is further iterated with a fixed step size. Even when the resultant force is zero, the algorithm continues iterating, effectively addressing local minimum and optimizing the iteration speed and randomness of the RRT algorithm. With the selection of the latest point $x_{new1}$, the combined RRT and potential field algorithm selects random points for iteration based on the nearest point. However, local minima can still obscure force directions, causing the iteration process to halt. The solution process of this shown in Figure 4.

After connecting the two trees, the algorithm attempts to reconnect all points more than five steps apart and uses an obstacle avoidance function to check for collisions.

## 5. Analysis of the FRRT*-Connect Algorithm

The effectiveness of trajectory planning algorithms can be evaluated through several key factors: success rate, path length, time complexity, and space complexity. The success rate indicates how well the algorithm reaches its endpoint in various environments, directly reflecting its effectiveness. Time complexity and space complexity measure computational efficiency, focusing on runtime and memory usage, respectively. These aspects can be analyzed using performance tools.

Additionally, path smoothness, robustness in dynamic environments, obstacle avoidance capabilities, applicability, and multi-objective optimization are crucial considerations. Simulations and real-world tests can validate these factors. User-friendliness is assessed by the ease of parameter adjustment and the intuitiveness of visualization tools, ensuring practical convenience and ease of debugging. Together, these evaluation methods provide a comprehensive understanding of the algorithm’s performance and reliability.

### 5.1. Probabilistic Completeness

The success of the algorithm depends on its ability to guide the robot effectively and safely to the target point while avoiding all obstacles. The probabilistic completeness of a trajectory planning algorithm means that if a path exists between the start and goal points, the algorithm will eventually find it given sufficient time or adequate sampling. Specifically, if the sampling is dense enough or the search time is long enough, the algorithm can find a feasible path in free space $x_{free}$. The sampling space  δ  is defined as the set of all valid sampling points, where each basic element $\delta_{n}$ is a sequence of n sampling points $\left\{x_{start},\ldots ,{\;x}_{goal}\right\}$ that form a collision-free path from $x_{start}$ to $x_{goal}$.

After discretizing the continuous path $\delta_{f}$ into an infinite set of sampling points, $\delta_{n}$ becomes a subset of $\delta_{f}$. Consequently, the sampling space δ can be viewed as the collection of all sequences of sampling points that form feasible paths. As the number of sampling points increases, δ progressively shrinks to an empty set, ensuring the discovery of a collision-free path composed of n sampling points, thereby addressing the path search problem.

Assuming that the $x_{start}$ and the $x_{goal}$ are connected and sampling is uniform, the set of connecting nodes V and the set of edges E must satisfy the conditions $V\subseteq {\;x}_{free}$ and $E\subseteq {\;x}_{free}$. As the number of sampling points increases, a set of nodes V can be identified to form sequence $\left\{x_{start},\ldots ,{\;x}_{goal}\right\}$.

Additionally, for bidirectional trees expanding from the $x_{start}$ and the $x_{goal}$, $T_{1}⊃(V_{1},E_{1})\subseteq {\;x}_{free}$ and $T_{2}⊃(V_{2},E_{2})\subseteq {\;x}_{free}$ are defined. Here, $V_{1}$ and $V_{2}$ represent the sets of all nodes in the two trees, while $E_{1}$ and $E_{2}$ denote the sets of edges connecting these nodes. In these two trees, nodes $V_{1i}\in V_{1}$ and $V_{2j}\in V_{2}$ must exist, such that the Euclidean distance between them is less than a threshold distance $dist\left(V_{1},V_{2}\right)\leq Thr$, ensuring that $\delta_{n}$ is included in $\left\{x_{start},\ldots ,V_{1i},V_{2j},\ldots ,x_{goal}\right\}$, and that $\delta_{n}\subseteq \delta_{f}$ is connected.

### 5.2. Asymptotic Optimality

Asymptotic optimality in trajectory planning algorithms refers to the property that as the algorithm’s runtime or number of iterations increases, the obtained path solution increasingly approaches the global optimal solution. During the iterative process, the trajectory generated by the algorithm progressively converges towards the optimal solution. In the study [26], the RRT* algorithm continuously optimizes the path generation and connection process by reconnecting the parent nodes of the latest point. Each iteration expands and adjusts based on the currently optimal path. By refining strategies such as node selection, path connection, and distance measurement, the RRT* algorithm can progressively improve the quality of the generated path, thus demonstrating its asymptotic optimality.

Given that there exists an optimal distance path $\delta_{best}$ from the $x_{start}$ to the $x_{goal}$, in this algorithm, the random tree $T_{1}$ extending from $x_{start}$ connects to a point $V_{1i}$ of the random tree $T_{2}$ extending from $x_{start}$. This segment of the path is defined as $\sigma_{f1}$. The path $\sigma_{f1}$ consists of a sequence of points from $x_{start}$ to $V_{1i}$, while the path $\sigma_{f2}$ consists of a sequence of points from $x_{goal}$ to $V_{2j}$. The entire path generated by the algorithm can be represented as.

(7) $$\sigma_{f\;}={\;\sigma }_{f1\;}+{\;\sigma }_{f2}+dist\left({\;V}_{1i}+V_{2j}\right)$$

In addition, this algorithm incorporates the principles of the RRT* algorithm by reconnecting the latest point with its parent nodes within a certain distance during each iteration. After linking the two trees, the algorithm also performs reconnections within a specified step size for the entire tree. This implies that, given a fixed initial position, each additional iteration will result in the following phenomena.

(8) $$\left\{\begin{array}{c} dist\left(\sigma_{f1\left(n+1\right),}n\right)\leq dist\left(\sigma_{f1\left(n\right)}\right)\\ dist\left(\sigma_{f2\left(n+1\right),}n\right)\leq dist\left(\sigma_{f2\left(n\right)}\right)\\ dist\left(T\right)\leq dist\left(T_{1}\right)+dist\left(T_{2}\right) \end{array}\right.$$

In Equation (7), $dist\left(\sigma_{f1\left(n+1\right),}n\right)$ represents the distance of the first n steps generated during the n+1 iteration of the path.

As the number of iterations n approaches infinity in the algorithm, the distance $dist\left(\sigma_{f}\right)$ of the path $\sigma_{f}$ will approach the distance $dist\left(\sigma_{best}\right)$ of the optimal path $\sigma_{best}$, as below:

(9) $${lim}_{n\to \infty }dist\left(\sigma_{f\left(n\right)}\right)\;\to \;dist\left(\sigma_{best}\right)$$

Based on the above reasoning, the FRRT*-Connect algorithm exhibits asymptotic optimality.

### 5.3. Time Complexity

Time complexity measures how the execution time of an algorithm increases with the size of the input. It reveals the amount of time required by the algorithm in the worst case and is typically expressed as a function of the input size n. Analyzing time complexity is crucial for evaluating the practical usability and efficiency of algorithms, especially when dealing with high-dimensional or complex environments.

For the FRRT*-Connect algorithm, as shown in the pseudocode diagram, the algorithm primarily consists of a single loop. The main steps of the algorithm include: random sampling Sample, finding the nearest point, computing attraction forces calcuGravitation, computing repulsion forces calcuReplusive, calculating the resultant force sumforce, expanding the random tree steer, performing collision detection CollisionFree, rewriting parent nodes rewrite, and reconnecting the entire tree after linking bestpath.

For the above steps, let the total time complexity of the algorithm be $T_{total}$, with individual time complexities denoted as $T_{sample},T_{near},T_{calcuGravitation},T_{calcuReplusive},T_{steer}$, ${T_{sumforce},T}_{CollisionFree},T_{rewrite},T_{bestpath}.$ Assume the number of iterations is N. In the space, random sampling $T_{sample}$ and the expansion step $T_{steer}$ typically do not depend on the number of tree nodes, and the time complexity of these two steps is O(N). The time complexity of the nearest neighbor search $T_{near}$ using a k_d tree is O(N∗logN). During the iteration process, the time complexity for computing attraction forces $T_{calcuGravitation}$ and repulsion forces $T_{calcuReplusive}$ depends on the iteration steps and the nodes of the tree, where the time complexity for attraction is O(N), and for repulsion is O(N+M), with M being the number of obstacles to be traversed. The time complexity for collision detection $T_{CollisionFree}$ depends on the spatial partitioning and the shape of obstacles, typically represented by O(N∗logN). The time complexity for rewriting parent nodes $T_{rewrite}$ and finding the shortest path point after completing the search $T_{bestpath}$ is typically represented by O(N) and O(1). Therefore, the total time complexity of the FRRT*-Connect algorithm can be expressed as:

(10) $$\begin{array}{c} T_{total}={\;T}_{sample}+T_{near}+T_{calcuGravitation}{+T}_{calcuReplusive}+T_{steer}\\ +{\;T_{sumforce}+T}_{CollisionFree}+T_{rewrite}+T_{bestpath}\;\\ \begin{array}{c} =O\left(N\right)+O\left(N*logN\right)+O\left(N\right)+O\left(N\right)+O\left(N+M\right)\\ +O\left(N*logN\right)+O\left(N\right)+O\left(1\right)\;\\ =2*O\left(N*logN\right)+4*O\left(N\right)+O\left(N+M\right)+O\left(1\right)\; \end{array} \end{array}$$

Since the number of iterations N is significantly larger than other minor factors, the overall time complexity is primarily determined by N. Therefore, the overall time complexity of the algorithm can be simplified to $O\left(N*logN\right)$.

(11) $$T_{total}\to O\left(N*logN\right)$$

The time complexity of the FRRT*-Connect algorithm is equivalent to that of the RRT-Connect and RRT*-Connect algorithms.

## 6. Algorithm Testing and Simulation

### 6.1. Test Analysis

This study proposes the FRRT*-Connect algorithm to enhance the search efficiency of RRT in three-dimensional space while overcoming the limitations of the artificial potential field (APF) in complex obstacle environments. This algorithm addresses the applicability challenges of the artificial potential field method in environments with singular obstacles, concave obstacles, narrow passages, and varying obstacle densities, thereby optimizing path planning capabilities.

To validate the adaptability of the FRRT*-Connect algorithm in complex three-dimensional spaces, this study constructs three types of 3D simulation environments based on the aforementioned obstacle characteristics.

Environment 1 (dense obstacle field): this simulation environment, measuring 1000 × 1000 × 1000 units, consists of 100 randomly distributed obstacles and includes three sub-environments: a spherical obstacle field, a cylindrical obstacle field, and a cuboidal obstacle field. As shown in Map 1, the radii of the spherical obstacles range from 50 to 70 units. In Map 2, the base radii of the cylindrical obstacles range from 50 to 70 units, with heights spanning 50 to 150 units. Map 3 illustrates cuboidal obstacles, with edge lengths ranging from 90 to 100 units. The minimum spacing is controlled by 20 units, creating a high-density spatial partition to evaluate the algorithm’s path planning performance in environments with multiple complex obstacles.

Environment 2 (maze obstacle field): this simulation environment measures 1000 × 1000 × 20 units and consists of two independent maze units. Map 4 illustrates a three-dimensional maze environment featuring dead-end traps, including three enclosed branch paths designed to simulate path misguidance in real-world scenarios. The maximum length of all navigable passages is 200 units. Map 5 further illustrates a twisting maze environment, featuring only a single navigable path designed to assess the algorithm’s ability to correct direction in misleading environments.

Environment 3 (narrow obstacle field): this simulation environment also measures 1000 × 1000 × 1000 units. The passage structure consists of two sets of cuboidal obstacles arranged in parallel (each measuring 499 × 600 × 1000 units), forming an extremely narrow gap of only two units in width, with no constraints in the vertical direction. Additionally, two cylindrical obstacles are placed at both ends of the passage to increase the complexity of the surrounding area. This environment aims to evaluate the algorithm’s adaptability and path planning capabilities in ultra-narrow passage conditions.

**Figure 6:**
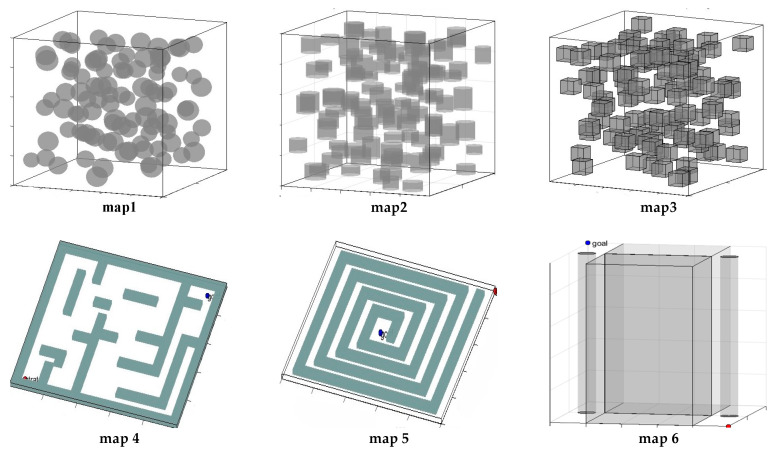
Schematic diagram of the barrier environment.

The construction of these three simulation environments effectively validates the improved performance of the FRRT*-Connect algorithm across various complex settings and provides a reliable benchmark for future research in path planning algorithms (Figure 6).

The experiments were conducted on a high-performance computing platform (Intel^®^ Core™ i7-8750H CPU @ 2.20 GHz and NVIDIA GeForce GTX 1060 GPU) to evaluate the performance of six path planning algorithms in three complex environments: dense obstacle environments, maze environments, and narrow passage environments. Each algorithm was executed independently 100 times in each environment, with success rate, path length, and computation time recorded. The compared algorithms included APF (Khatib, 1986) [9], RRT (Karaman, 2001) [7], RRT*-Connect (Klemm, 2015) [27], Informed-RRT* (Gammell et al., 2014) [28], AEB-RRT (Wang, 2022) [29], and the proposed FRRT*-Connect algorithm. The evaluation focused on key performance metrics such as path quality, convergence speed, and success rate to validate the overall superiority of FRRT*-Connect in complex environments.

**Figure 7:**
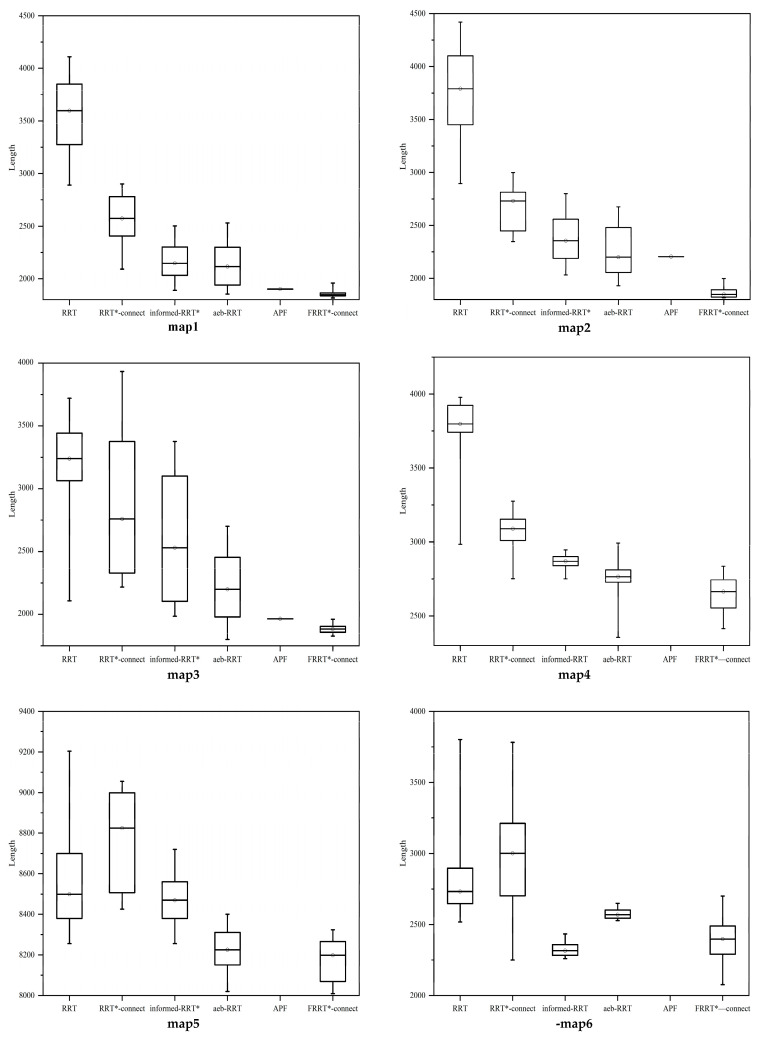
Path lengths of each algorithm in 3D environments.

**Figure 8:**
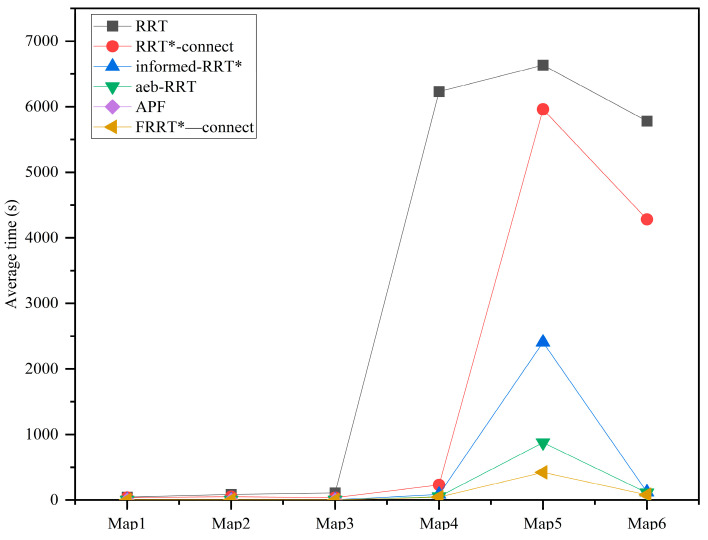
Average running times.

The results of the comparative simulation experiments are illustrated in Figure 7, where each algorithm was executed 50 times in a 3D environment. The iteration count for each trial was limited to 100,000; if no valid path was found within this limit, the attempt was considered a failure. The obtained path lengths were visualized using box plots. The step size and connection threshold for all algorithms were set to 15 and 30 units, respectively. The average execution times across different environments are summarized in Figure 8, while the failure rates of path searches are presented in Table 2. The simulation result demonstrates that, across all tested environments, the direction-aware FRRT*-Connect* algorithm consistently exhibited superior path–search efficiency while significantly reducing computational time compared to other methods.

The simulation results demonstrate that the FRRT*-Connect algorithm exhibits superior path search performance across six distinct test environments. In three-dimensional settings, the algorithm outperforms most sampling-based algorithms in both search rate and success rate, especially in the complex environments depicted in Figure 7 with map1–map3, where its search speed is second only to that of the artificial potential field (APF) algorithm. However, as shown in Figure 7 with map4–map6, the APF algorithm tends to become stuck in local minima in narrow obstacle regions and complex maze environments, resulting in ineffective results in these challenging scenarios. In contrast, FRRT*-Connect mitigates this issue by optimizing the sampling strategy and utilizing a bidirectional search mechanism, successfully finding paths even in challenging environments.

According to the simulation result, the Informed-RRT* algorithm, constrained by its elliptical sampling region, demonstrates relatively fast search capabilities in certain environments. However, in maze-like environments, the algorithm can experience significant delays due to issues such as dead-ends and overlapping sampling regions, thereby affecting search efficiency. In comparison, FRRT*-Connect enhances sampling efficiency by improving its sampling strategy and bidirectional search mechanism, ensuring the optimality of collision-free paths while increasing search efficiency. The optimized sampling approach effectively limits the search space, further accelerating the sampling process. Additionally, the algorithm incorporates a dynamic adjustment of the APF’s potential field, enabling adaptive recalibration of the gravitational field when a search tree encounters local minimum, thereby circumventing suboptimal solutions. This mechanism not only accelerates the sampling process and improves the accuracy of pathfinding but also addresses the limitations of the traditional APF algorithm in complex obstacle-rich environments, further enhancing the algorithm’s adaptability and stability in challenging environments.

To provide a clear illustration of the execution time of each algorithm, a detailed schedule of their runtime is presented below.

To comprehensively evaluate the geometric quality of the planned trajectories, we introduce two quantitative metrics: cumulative curvature variation (*CCV*) and continuity index ($C^{1}$). These indicators, respectively, reflect the smoothness and directional stability of a trajectory, which are crucial for ensuring feasible and stable execution in robotic systems. The *CCV* measures the total angular deviation along a discretized path. For a trajectory represented by n consecutive points $\left\{p_{1},p_{2},p_{3},\ldots ,p_{n}\right\}$, the local turning angle ∅ at each interior point is calculated as:

(12) $$\emptyset_{i}=\arccos\left(\frac{\left(p_{i+1}-p_{i}\right)\cdot \left(p_{i+2}-p_{i+1}\right)}{\left|\left|p_{i+1}-p_{i}\right|\right|\cdot \left|\left|p_{i+2}-p_{i+1}\right|\right|}\right)$$

The cumulative curvature variation is then defined as the sum of all such angle changes:

(13) $$CCV=\sum_{i=1}^{n-2}\left|\emptyset_{i}\right|$$

A lower *CCV* value indicates a smoother trajectory with fewer sharp turns.

The $C^{1}$ continuity index reflects the variation in discrete velocities along the path. With $v_{i}=||{\vec{p}}_{i+1}-{\vec{p}}_{i}||$, we define the local velocity change as $\delta_{i}=\left|\left|{\vec{v}}_{i+1}-{\vec{v}}_{i}\right|\right|$. Normalizing over the maximum velocity magnitude $v_{max}$, the $C^{1}$ continuity index is defined as:

(14) $$C^{1}=1-\frac{1}{n-2}\sum_{i=1}^{n-2}\min\left(1,\frac{\delta_{i}}{v_{max}}\right)$$

This index ranges from 0 to 1, with higher values indicating smoother and more continuous velocity profiles, which are particularly desirable for robotic systems with dynamic constraints.

In comparative experiments, We take the average of the 50 run results for maps 1, 2, and 3, respectively. The proposed FRRT*-Connect algorithm achieved a lower CCV (64 radians) and a higher $C^{1}$ continuity index (0.9033) than the standard RRT*-Connect (104 radians and 0.6457, respectively). These results verify the algorithm’s ability to produce both directionally and kinematically smoother trajectories.

In this study, the proposed FRRT-Connect* algorithm outperforms five other mainstream path planning algorithms, including APF, RRT, RRT*-Connect, Informed-RRT*, and AEB-RRT, in complex environments. Compared to traditional RRT and RRT*-Connect, FRRT*-Connect significantly improves path smoothness and continuity by optimizing the sampling strategy and introducing a bidirectional search mechanism, while effectively avoiding the local minimum problem. In multiple test environments, FRRT*-Connect not only leads in search efficiency, but also demonstrates superior performance in path quality and adaptability, especially in high-density obstacle and narrow passage environments. Overall, FRRT*-Connect provides a more stable and reliable path planning solution in complex obstacle-rich environments.

### 6.2. Algorithm Simulation

The FRRT*-Connect algorithm is simulated using a 7-DOF Kinova Gen3 robotic arm to conduct kinematic tests. This aims to assess the algorithm’s effectiveness and performance in obstacle avoidance for the robotic end-effector.

The robotic arm is illustrated in Figure 8. The simulation environment is MATLAB 2022a, with both the base coordinates and end-effector of the robotic arm marked. The kinematic solutions are obtained using the Denavit–Hartenberg parameters provided in the Kinova robotic arm’s documentation, as illustrated in Table 3.

Several case simulations are conducted to evaluate the algorithm’s effectiveness in obstacle avoidance for the end-effector under various obstacle conditions.

**Figure 9:**
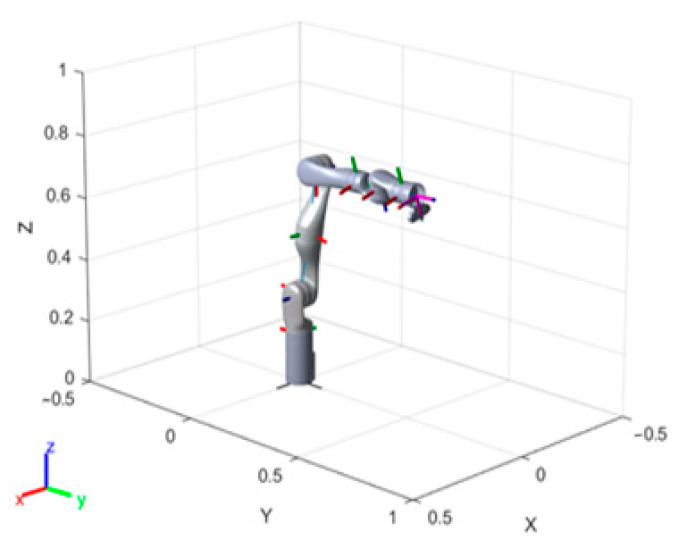
7-DOF Kinova Gen3 robotic arm diagram.

The initial position of the function is determined by solving the inverse kinematics for the current position, with the initial joint angles being [−49.38°, −77.36°, 0.000000096°, 0.0000000018°, −86.35°, −1.91°, 0.00°]. The start point is set at [−0.6, −0.75, 0.5] and the end point is set at [0.6, −0.75, 0.5]. Three types of obstacles are introduced between these two points, and the following examples are proposed. The structural model of the robotic arm and the Denavit–Hartenberg (DH) parameters corresponding to its initial position are presented in Figure 9 and Table 4, respectively.

**Figure 10:**
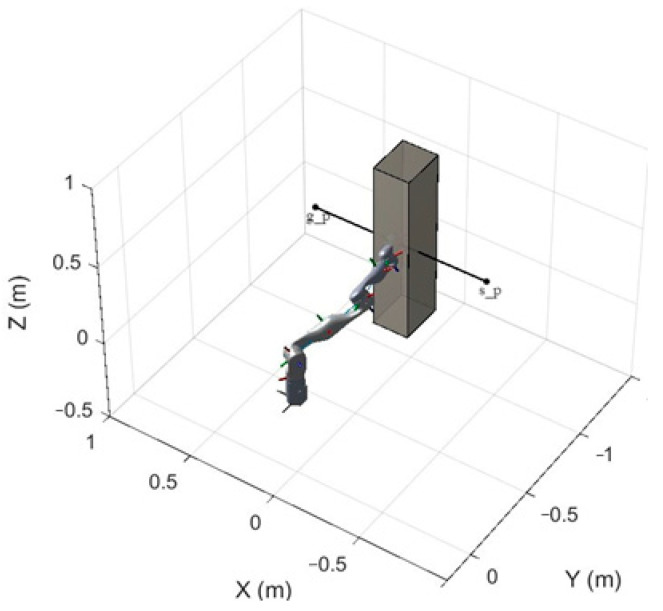
The traditional APF algorithm generates a path when encountering a rectangular prism obstacle.

First, the performance of the artificial potential field (APF) algorithm in handling complex obstacle environments is presented, particularly when these obstacles exclude point obstacles. This section provides a detailed analysis of the algorithm’s effectiveness and limitations when dealing with various complex obstacle shapes and layouts. By comparing path planning results under different obstacle configurations, we reveal the performance characteristics of the algorithm in complex environments, including its ability to generate paths through dynamic and irregular obstacles. These analyses provide valuable insights for further optimizing the algorithm, helping to identify areas for improvement to address challenges and limitations in practical applications. The simulation results obtained using the APF algorithm are shown in Figure 10.

In path planning using artificial potential field methods, gradient disappearance and local minima are pervasive and challenging issues. These problems significantly impact the effectiveness and reliability of the algorithm, particularly in complex environments. The gradient disappearance problem can result in insufficient guidance near the target in the potential field, preventing the algorithm from effectively escaping local regions, thus limiting its ability to find a global optimal path. These issues severely restrict the effectiveness of artificial potential field methods in complex obstacle environments, especially in scenarios with multiple obstacles, complex shapes, or dynamic changes.

**Figure 11:**
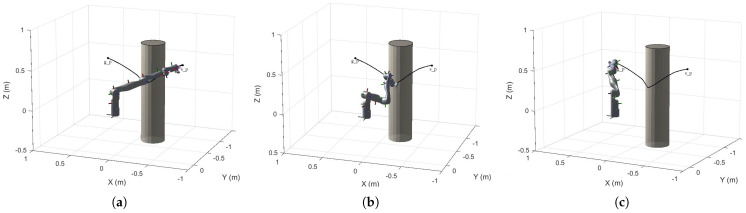
(**a**) Initial pose of the robotic arm; (**b**) pose of the robotic arm with the potential field function applied for obstacle avoidance; (**c**) final pose of the robotic arm.

We introduce an improved strategy by combining the RRT*-Connect path planner. The RRT*-Connect algorithm has global optimization capabilities and can provide more comprehensive path exploration in complex environments. By combining RRT*-Connect with the artificial potential field method, we can effectively alleviate the issues of gradient vanishing and local minima, thereby improving the quality and effectiveness of path planning. This combination allows the algorithm to retain the fast search speed of the artificial potential field method while leveraging RRT*-Connect to overcome local minima. The simulation results for the cylindrical obstacle are provided in Figure 11.

**Figure 12:**
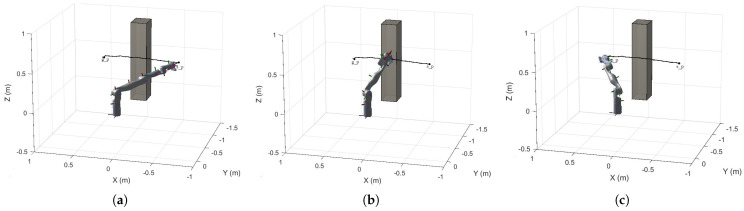
(**a**) Initial pose of the robotic arm; (**b**) pose of the robotic arm with the potential field function applied for obstacle avoidance; (**c**) final pose of the robotic arm.

In Example 1, we used a repulsive function for path planning around cylindrical obstacles to improve obstacle avoidance. Adjusting the attraction and repulsion coefficients effectively controlled the robot arm’s path and response, significantly shortening the path length. This approach also addresses the local minima problem of traditional potential field methods, enhancing stability and reliability in complex environments. The following example illustrates the application of potential field functions for handling cuboid obstacles. The simulation results for the cuboid obstacle are provided in Figure 12.

The simulation result confirms the effective integration of the RRT*-Connect algorithm with the artificial potential field method. The global optimization features of the RRT*-Connect algorithm significantly alleviate the issue of local minimal inherent traditional artificial potential field methods, allowing the robotic arm to generate more optimal paths in complex environments. The artificial potential field method, on the other hand, offers a notable advantage in path search speed, especially when handling dynamic and intricate obstacles.

## 7. Conclusions

The proposed FRRT*-Connect algorithm effectively addresses the local minimum issue in three-dimensional environments with traditional artificial potential field methods and the low-efficiency issue of traditional RRT algorithms in narrow passages. Compared to other classic algorithms, FRRT*-Connect demonstrates faster path planning speed and higher precision in complex environments, as well as significant advantages in end-effector obstacle avoidance. By introducing an attractive force function and biased sampling strategy, the algorithm generates optimized paths in a shorter time while maintaining the same time complexity as traditional methods. Specific improvements include enhancing the modeling of repulsive potential fields to reduce path wastage, introducing dynamic attractive force control to effectively overcome local minima, and optimizing the path after generation to ensure the shortest path. Simulation results show that FRRT*-Connect significantly improves path planning efficiency, accuracy, and success rate in complex environments, especially in narrow passage scenarios, with a success rate of 98%. These improvements effectively enhance the algorithm’s adaptability and robustness, validating its effectiveness in obstacle avoidance.

Although this study has significantly improved the performance of path planning algorithms in complex environments, several challenges remain. First, the computational efficiency of the algorithm in high-dimensional environments requires further optimization to broaden its applicability. Second, the algorithm’s adaptability, particularly in handling complex topologies and dynamic environments, needs enhancement. Lastly, path post-processing can be further refined to reduce path length and improve smoothness. Future research will primarily focus on addressing these challenges, with a particular emphasis on dynamic environments, to extend the algorithm’s versatility and robustness.

## Figures and Tables

**Table 1:** Selection of the repulsive coefficient k.

Distance (m)	0–346	346–692	692–1039	1039–1385	1385–1732
k	1211	2431	3651	4881	6101

**Table 2:** Average running timing.

Algorithm	rrt (s)	rrt*-Connect (s)	Informed-rrt* (s)	Aeb-rrt* (s)	APF (s)	Frrt*-Connect (s)
Map1	47.5	40.9	2.01	1.79	0.89	1.49
Map2	86.3	52	2.95	1.78	0.68	1.243
Map3	111	37.9	4.79	2.75	0.86	1.5596
Map4	6231	231	86.7	52.7	-	45.7
Map5	6634	5962	2406	876	-	422.8
Map6	5782	4283	120.86	110.6	-	81.07

**Table 3:** Operation failure rate.

Algorithm	rrt	rrt*-Connect	Informed-rrt*	Aeb-rrt*	APF	Frrt*-Connect
Map1	0	0	0	0	0	0
Map2	0	0	0	0	0	0
Map3	2%	0	0	0	0	0
Map4	46%	24%	42%	0	100%	0
Map5	56%	46%	18%	0	100%	0
Map6	62%	56%	12%	2%	100%	2%

**Table 4:** Denavit–Hartenberg parameters corresponding to 7-DOF Kinova Gen3 robotic arm.

joint	α (rad)	d (m)	θ (rad)	r (m)
1	π/2	−0.1284	$\theta_{1}$	0
2	−π/2	−0.0064	$\theta_{2}$	0
3	π/2	−0.2104	$\theta_{3}$	0
4	−π/2	−0.0064	$\theta_{4}$	0
5	π/2	−0.1059	$\theta_{5}$	0
6	−π/2	0	$\theta_{6}$	0
7	π	−0.0615	$\theta_{7}$	0

## Data Availability

Data are contained within the article.
